# Factors Affecting Citizen Trust and Public Engagement Relating to the Generation and Use of Real-World Evidence in Healthcare

**DOI:** 10.3390/ijerph19031674

**Published:** 2022-02-01

**Authors:** Denis Horgan, Bettina Borisch, Ivana Cattaneo, Mark Caulfield, Arturo Chiti, Christine Chomienne, Amanda Cole, Karen Facey, Allan Hackshaw, Minna Hendolin, Nadia Georges, Dipak Kalra, Birutė Tumienė, Martina von Meyenn

**Affiliations:** 1 European Alliance for Personalised Medicine, 1040 Brussels, Belgium; 2 Department of Histopathology, University of Geneva, 1202 Geneva, Switzerland; Bettina.Borisch@unige.ch; 3 Novartis Farma SpA, 21040 Origgio, Italy; ivana.cattaneo@novartis.com; 4 NIHR Barts Biomedical Research Centre, William Harvey Research Institute, Queen Mary University of London, London EC1M 6BQ, UK; m.j.caulfield@qmul.ac.uk; 5 Department of Biomedical Sciences, Humanitas University, Via Rita Levi Montalcini 4, Pieve Emanuele, 20090 Milan, Italy; arturo.chiti@hunimed.eu; 6 IRCCS Humanitas Research Hospital, Via Manzoni 56, Rozzano, 20089 Milan, Italy; 7 Cell Biology-Hematology Department, Université de Paris INSERM, 75010 Paris, France; chomienne.christine@gmail.com; 8 Office of Health Economics, London SW1E 6QT, UK; acole@ohe.org; 9 Usher Institute of Population Health Sciences and Informatics, University of Edinburgh, Edinburgh EH8 9YL, UK; karen.facey@ed.ac.uk; 10 Faculty of Medical Sciences, University College London, London W1T 4TJ, UK; a.hackshaw@ucl.ac.uk; 11 Sitra—The Finnish Innovation Fund, 00180 Helsinki, Finland; minna.hendolin@sitra.fi; 12 Exact Sciences, 1201 Geneva, Switzerland; ngeorges@exactsciences.com; 13 Faculty of Medicine and Health Sciences, University of Ghent, 9000 Ghent, Belgium; dipak.kalra@i-hd.eu; 14 Vilnius University Hospital Santariskiu Klinikos, 08661 Vilnius, Lithuania; birute.tumiene@santa.lt; 15 F. Hoffmann-La Roche AG, 4070 Basel, Switzerland; martina.von_meyenn@roche.com

**Keywords:** citizen trust, real-world evidence, real-world data, evidence framework

## Abstract

The potential for the use of real-world data (RWD) to generate real-world evidence (RWE) that can inform clinical decision-making and health policy is increasingly recognized, albeit with hesitancy in some circles. If used appropriately, the rapidly expanding wealth of health data could improve healthcare research, delivery of care, and patient outcomes. However, this depends on two key factors: (1) building structures that increase the confidence and willingness of European Union (EU) citizens to permit the collection and use of their data, and (2) development of EU health policy to support and shape data collection infrastructures, methodologies, transmission, and use. The great potential for use of RWE in healthcare improvement merits careful exploration of the drivers of, and challenges preventing, efficient RWD curation. Literature-based research was performed to identify relevant themes and discussion topics for two sets of expert panels, organized by the European Alliance for Personalised Medicine. These expert panels discussed steps that would enable a gradual but steady growth in the quantity, quality, and beneficial deployment of RWE. Participants were selected to provide insight based on their professional medical, economic, patient, industry, or governmental experience. Here, we propose a framework that addresses public trust and access to data, cross-border governance, alignment of evidence frameworks, and demonstrable improvements in healthcare decisions. We also discuss key case studies that support these recommendations, in accordance with the discussions at the expert panels.

## 1. Introduction

Major innovation in healthcare systems is often shaped by crises rather than strategic planning, as demonstrated by the recent explosion in data science during the COVID-19 pandemic [1]. Although the pandemic has posed multiple challenges, it has also catalyzed progressive adoption of data and digital health policies forced by the pandemic-driven urgency to accelerate digitalization of healthcare systems and public awareness of the utility of patient-level data. Such real-world data (RWD), defined by the European Medicines Agency (EMA) as “routinely collected data relating to a patient’s health status or the delivery of healthcare from a variety of sources other than traditional clinical trials” [2], can be obtained from a range of sources, including electronic health records, patient registries, and wearable and mobile technologies [3]. Interoperable and high-quality RWD, as well as a proportionate governance framework for their use, is needed to generate real-world evidence (RWE), which is critical for improving the understanding of the natural course of different diseases and of the benefit–risk profile of new and existing therapies, disease management, and healthcare provision. In addition, it provides valuable insights into clinical decision-making and healthcare resource use and informs future development programs. In Europe, healthcare regulatory authorities, health technology assessment (HTA) agencies, insurers, economists, medical and scientific societies, and pharmaceutical companies, increasingly rely on and make use of RWE in addition to traditional clinical trials to inform research and treatment development, regulatory decision-making, reimbursement decisions, and the development and implementation of evidence-based resources, including clinical practice guidelines, care pathways, and algorithms [2,4,5]. Notably, the development of data governance structures and HTA processes for the use of RWE have been greatly supported by patient advocacy groups, which have facilitated awareness of and education around how these kinds of RWD can help to improve patient outcomes (for example, through public–private partnership initiatives) [6].

Nevertheless, numerous challenges still limit the use of RWD, notably in terms of poor data capture, low data quality, and lack of interoperability, as well as low levels of consensus on methodologies for data collection, sharing, analysis, and reporting, and a still-limited understanding of RWD use. In addition, there is widespread hesitancy to accept the significance of RWD as evidence, to find sensible pathways guiding appropriate methodologies and quality criteria that take the current RWD landscape and these challenges into account, and to react accordingly. Aptly channeled, this experience could lead to wider acceptance and a more rapid exploitation of RWE generation as a resource for improved health and better healthcare provision in areas of Europe, with a reach far wider than the specifics of the COVID-19 pandemic. This, however, requires an additional level of strategic thinking, which is what this article aims to offer.

### 1.1. Value of RWD

RWD can provide many valuable insights into the patient experience as well as information to support drug development by allowing detection of unexpected outcomes, with the added benefit that generation of RWE is potentially faster than evidence from clinical trials and without the bias imparted by certain study designs [7]. From a research perspective, RWD can be used to examine trends in patient outcomes over time, to compare patient outcomes between different regions or countries, to examine patients’ experience of drugs (including adherence and quality of life), and to find ways of predicting patient outcomes, such as progression and survival. Prior to a drug being licensed, RWD can be used to provide a comparator for single-arm trials of experimental therapies [8], which is particularly useful for rare cancers and subtypes, as well as other rare diseases. RWD can also be used in a post-marketing setting to examine adverse events in a large patient population to confirm the safety profile of the drug, to find signals of uncommon side effects, to check that the effectiveness of the drug in routine clinical practice is similar to that seen in randomized controlled trials (RCTs), and to obtain evidence of drug efficacy and safety in subgroups of patients not included in the pivotal RCTs, so that the license can be extended to these subgroups [8].

### 1.2. Challenges to the Implementation of RWE

Evidence-based decision-making has been promoted for decades and relies on traditional evidence hierarchies. Although the value of timely, accurate health system data to inform public health and health system operations and social policy has long been understood, the pandemic has served to highlight the critical importance of its adoption. The question, therefore, is, “How can health systems and EU policy makers learn from this experience to build more resilient and efficient healthcare systems?” This must address heterogeneity in the provision and upgrading of healthcare and data infrastructures across Europe and address barriers and challenges to RWE implementation, including:

- Methodological issues: Most health data are captured routinely to serve a narrow purpose. Despite guidelines regarding data management (e.g., the FAIR [Findable, Accessible, Interoperable and Reusable] Principles on data management) [9], there is no overall consensus on data collection for RWE studies. In addition, there is limited guidance outside of clinical trials or studies to specify the type of data that should be captured, i.e., minimal datasets in a routine clinical setting and the definition of clinically meaningful data. This lack of data standardization, completeness, quality assurance, and accessibility amounts to a serious limitation on comparison and pooling of data for different datasets and the usefulness of collected data for answering specific scientific and medical questions [4,10].
- Lack of harmonization between RWD collection systems: Deriving useful insights from the sharing of data between different centers or countries is possible only through data collection harmonization, data transparency, and endorsement of collection methods within individual countries and a common interest in answering a specific question with the collected data.
- Data-access and data-sharing limitations: Data sharing between countries is subject to further practical challenges, often because the EU General Data Protection Regulation (GDPR) impedes easy understanding of how to exchange data for research. Despite the harmonizing intentions of GDPR, significant variations remain at national levels in interpretations of this legislation.
- Limitations of regulatory agencies and HTAs/payers: Attitudes toward use of RWD vary across Europe, with differing degrees of acceptance for its different policy areas and differing expertise to critically assess its use in agencies’ decisions and recommendations [11]. Guidance provided by agencies often reflects the ideal end-stage for RWD, where data are available in sufficient quantities and are of sufficient quality. Efforts are needed to guide stakeholders in this learning healthcare ecosystem state to use what is available appropriately.
- Lack of citizen trust in data sharing: RWD can be obtained from either regional or national databases, where researchers can be granted access to the data, or from sponsored RWD studies, which require informed consent for data collection from participating patients. The former databases are obligated to ensure both data security and patient confidentiality. Widespread suspicion within the general population, a reluctance to surrender data to governments or multinational corporations, and concerns over cybersecurity hinder the sharing of patient data that could advance medical research. To circumvent these issues, patient involvement in the collection of RWD is crucial. The French COVID-19 Corona Tracker had a successful uptake, while German citizens were broadly resistant to their own country’s version. It is notable that the French model for data access provided patients with tangible outcomes from sharing their information, in a similar manner to the EU Patient Cancer Digital Centre/Platform, while the German system focused on tracking only and did not offer citizens much in terms of benefits or services.

In view of these challenges, the European Alliance for Personalised Medicine (EAPM) conducted a series of expert panels to assess the factors that affect public trust and engagement in the implementation of RWE across Europe. Based on these discussions, we aim to provide an overview of key use cases and initiatives that address these barriers and to outline a framework to facilitate implementation of RWE at country-specific and EU-wide levels. Below, we discuss the outcomes of these expert panels; however, we also aim to provide a preliminary review of the challenges and successes associated with establishing a coherent approach to health policy within the EU, since strategic thinking, rather than infrastructure or the software associated with data handling, is the key to incorporating RWE to improve healthcare.

### 1.3. Coherence in EU Health Policy Making

Policies to promote a uniform approach to RWD collection methodologies, harmonization of RWD collection systems, data access and sharing, use of RWD by regulatory agencies and HTAs/payers, and RWD security and confidentiality across the EU would greatly improve generation of RWE. However, there are competing views over the extent to which the EU could and should develop a closer engagement with Member States on health policy. Multiple initiatives have been proposed to align health policy across the EU, including a bid by the European Commission to bring coherence to the multiple HTA procedures across its Member States [12], the announcement of the creation of the European Health Union (EHU) [13], new measures prompted by the COVID-19 pandemic (ranging from strengthening the EMA and the European Centre for Disease Prevention and Control), to the creation of new structures to ensure cross-border coordination. Chronic drug shortages over recent years, resentment over high prices for innovative drugs, and most recently the inconsistent responses to the COVID-19 pandemic have led to admissions on all sides that current arrangements are insufficient to cope with changing healthcare challenges. Unfortunately, key stakeholders do not agree on the best approach to tackling these issues, with discussions regarding demands for additional harmonization of health policies being accompanied by a similarly vocal rejection of centralization of power [14].

Health policy was deliberately retained from the outset of the EU as a national competence by Member States and was mentioned in the founding treaties [15], although this was mainly to limit freedom of movement of workers or goods. Despite several updates and mentions of health in official EU documents, limitations are clear: “The EU does not define health policies, nor the organization and provision of health services and medical care” [16] and its role is “to complement national policies” [17]. The lack of overall accountability and responsibility for health continues to hinder EU discussions and has impacted the formulation of a coherent policy. Due to the complexity of personalized medicine, policy making decisions that may impact citizen trust or maximize the potential of RWE have proven challenging [18]. Even for medicines with a more stable framework within the context of the single market, there are difficulties with regulation and EU uncertainties. Thus, ensuring evidence-based innovative medical research is challenging and it is important that it does not limit improvements to healthcare and citizen health. Strategic thinking regarding the current gaps for ensuring trust in the use of citizen data for RWE is required [19].

## 2. Materials and Methods

**Figure 1:**
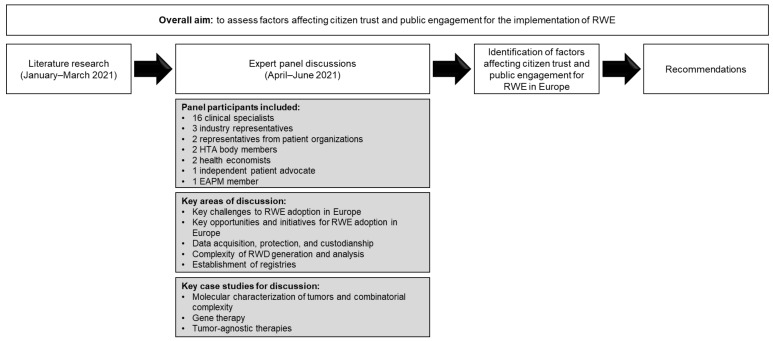
Methodology used to assess the factors affecting citizen trust and public engagement relating to the generation and use of RWE in healthcare. EAPM, European Alliance for Personalised Medicine; HTA, health technology assessment; RWD, real-world data; RWE, real-world evidence.

The methodology used to assess the factors affecting citizen trust and public engagement in RWE implementation is shown in Figure 1. Briefly, initial literature-based research was carried out to develop a framework to contextualize the issues relating to RWE, citizen trust, and public engagement within known barriers of access to personalized medicine. A literature review was performed using PubMed, Web of Science, and Medline, with search terms focusing on (synonyms of): ‘barriers’, ‘personalized (medicine)’, ‘NGS’, ‘treatment’, ‘Europe’, ‘patient’, ‘ethics’, ‘early diagnosis’, ‘clinical’, ‘public health’, ‘regulatory’, ‘legislation’, ‘trust’, ‘citizen engagement’, and ‘governance’. A total of 1343 English-language abstracts published between January 2015 and December 2021 were identified across all search engines. Searches restricted to publications from any EU country, or the UK, were also performed; however, these searches identified few records. Review of the results from search terms with large numbers of records resulted in 483 identified publications, which were screened for relevance by reading the title and abstract. Removal of duplicate records within and between search engines resulted in 73 remaining publications, which predominantly included articles related to ‘barriers’ and ‘personalized medicine’. These publications were used to identify current domains relevant to RWD for discussion in the expert panels. Two sets of expert panels involving participants who were selected to provide insight based on their expertise in medical, economic, patient, industry, or governmental (such as value access bodies) areas were held to discuss key areas of interest. The first set of expert panels were held on 27, 28, and 30 April 2021, with the second set held on 8–11 June 2021. The expert panels were carried out in three sessions (2.5 h each) over the course of 2 weeks, all following the same format and chaired by Denis Horgan, Executive Director of the EAPM. The panels included a mix of experts from different specialties involved with RWE generation (16 clinical specialists, three industry representatives, two representatives from patient organizations, two HTA body members, two health economists, one independent patient advocate, and one EAPM member) from 14 countries (Belgium, Bulgaria, Croatia, Finland, France, Germany, Italy, Lithuania, the Netherlands, Portugal, Spain, Sweden, Switzerland, and the UK). Participants were asked to provide input based on their own experiences and individual case studies were discussed to highlight areas where policy could be developed. The participant list and format for both series of expert panels were similar, although fewer participants attended the second set of panel discussions, based on experts’ ability to contribute substantially. Summary minutes were generated from each panel, and these were used to identify common themes and factors affecting citizen trust and public engagement in RWE across Europe. Based on the expert panel discussions, recommendations for best practice implementation were generated. Key case studies deemed to be the most relevant and supportive of these recommendations, in accordance with the outputs from the expert panels, are discussed.

## 3. Results

Although there was diverse discussion on RWE, citizen trust, and public engagement in the publications identified by the literature review, all publications highlighted the importance and interconnectivity of these issues. Many publications concluded that barriers related to citizen trust and public engagement in personalized medicine could be overcome as more data demonstrating its success become available. The uptake of personalized medicine is considered complex in that it requires a change not only in clinical practice, but also in the types of information required (i.e., the characteristics of the additional data and the information delivery that healthcare professionals and citizens would need to employ). Introduction of new instruments to personalize healthcare was not typically considered a complexity, as healthcare professionals and patients have a history of trying to individualize their treatment. Therefore, citizen trust and public engagement were deemed to be the most crucial issues. Striving for increased citizen trust and public engagement may therefore lead to better individual patient outcomes, resulting in improvements in the quality of healthcare, and a decrease in healthcare costs. To realize these benefits, the progression from early diagnosis to treatment and survivorship needs to overcome scientific (e.g., evidence and methodology), operational (e.g., regulations and information delivery), and economic (e.g., reimbursement and incentives) barriers. Personalized medicine will require changes in healthcare infrastructure, diagnostic models, and reimbursement policies.

Detailed results from the expert panels can be found in the “Discussion” section; however, the overarching principles from the expert panel discussions can be summarized as follows:

The guidelines on which clinical decisions are made are generally based around evidence from RCTs or meta-analyses of RCT data. Incorporation of RWE within clinical guidelines is a fundamental challenge that once addressed will provide a broader evidence base to support clinical decision-making. A separate issue is that lack of physical access to the appropriate guidelines within the consultation room or other clinical settings limits the use of evidence (real-world or otherwise) in clinical decisions. A solution to this could be to incorporate clinical guidelines into electronic health record systems so that the information can be delivered to clinicians in a context that is sensitive to individual patients. Both of these factors mean that, in reality, RWE—despite its advantages—rarely informs patient diagnosis or treatment choice. Availability of RWE for incorporation into clinical guidelines is impeded by a chain of related factors: insufficient access to data—often resulting from disparate national arrangements for its collection and transmission—and by lack of public conviction on the adequacy of those arrangements. These gaps are compounded by a lack of infrastructure and governance, and the absence of agreed methodologies for RWE assessment.

Analysis by the expert panel divided the challenge into four specific areas (Table 1): (1) promoting better access to data—a process that requires both personal data security and confidentiality—to be in a better position to generate RWE; (2) establishing a framework for generating RWE so that the methodology can be used to analyze data and create the evidence required to promote trust in RWE; (3) embedding RWE into decision-making, within and across borders; and (4) enabling patients and other members of the public to contribute to the decision-making based on access to their data. However, each of these aspects contain its own layers of responsibility.

### 3.1. Public Trust and Access to Data

The optimal framework would aim to provide transnational access to (and public trust in) RWE. That, however, would be dependent upon an EU-level governance framework for evidence to be developed and accepted, in a way that can be endorsed by EU regulators, policy makers at the Member State level, and payers. This, in turn, is only possible when national arrangements are also aligned with EU regulations and where early dialogue with regulators and payers is based upon consistent prioritization of evidence challenges. To effect full coherence, regional and local rules would also have to accord with national regulations, with agreements on inter-regional sharing of data.

### 3.2. Evidence Framework

An optimal evidence framework ultimately depends upon an agreed methodology, approved endpoints, adaptability to enable growth into new and evolving areas, and commitment for decision makers to assess RWE, employing data from clinical centers and research institutions that are federated at the regional level and subsequently organized at the national level. At the European level, when gathering information on national experiences, methods, and citizens’ involvement, this would provide a mechanism for the use of RWE that is trusted by the medical community and the public as well as guidance for evidence generators regarding the standards (e.g., disease stage, outcome measures, target population, and data-sharing requirements) required for RWD-enabled decision-making, while at the same time supporting clinical and reimbursement decisions and promoting innovation. It would also require demonstration of global improvement of healthcare and reduced costs.

### 3.3. Cross-Border Governance Framework to Facilitate RWE Decision-Making

Similarly, a cross-border governance framework to facilitate RWE decision-making presupposes early dialogue with regulators and payers, as well as established infrastructure, guidelines, and common global standards. The only feasible mechanism to attain this goal is an EU health governance agreement on common standards and evidence requirements for clinical and reimbursement decisions. The primacy of Member States’ sovereignty over such matters requires an upstream federation of national infrastructure to enable collection, use, and interpretation of data for these decisions. This is again reliant on regional infrastructure to reuse genomics and health data for RWE decision-making and on functioning national infrastructures or governance for adoption of RWE and for promoting understanding of its role among healthcare authorities.

### 3.4. Citizens—Improvements in Healthcare Decisions

From the perspective of citizens as the end users of healthcare, quality of life should improve where evidence ensures rational allocation of resources for prevention, early diagnosis, and improved healthcare system efficiencies. This would represent a major advance on the current situation, where RWE is still rarely taken into consideration. At local and regional levels, patients and citizens would benefit from the utilization and sharing of RWE, particularly if standards are agreed for evidence alignment so that data are shared at the national level. In that scenario, there is an incentive for healthcare actors, patients, and citizens across the EU to enthusiastically endorse use of a shared evidence framework for RWE to secure better prevention, diagnostic, and treatment decisions.

To a greater or lesser extent, the expert panels concluded that the conditions outlined in these distinct layers of responsibility are currently absent or lacking visible efficiency. Focused energies need to be directed at the disconnects to incorporate the potential of RWD and RWE into European care.

## 4. Discussion

### 4.1. Health Policy within the EU

Across Europe, the European Commission aims to support the protection and improvement of citizen health and seeks to support healthcare systems through legislation, financial support, best-practice facilitation and sharing, and activities promoting health [20]. The current priorities for the European Commission include several initiatives: building a strong EHU, protecting citizen health, providing support for tackling future health crises, and improving healthcare system resilience [21]. As indicated above, the scope of the actions that might be taken under this initiative remain under question, due to divergent views within the EU institutions responsible for legislation.

A common European Health Data Space (EHDS) could support access to different types of health data, with the aim of supporting healthcare delivery, research and policy making [22]. Specifically, this data system is intended to support Member States with data governance and data exchange, data quality, and infrastructure and interoperability [22]. Although proposals are expected, the European Commission has already indicated that data protection will have a high priority within its provisions. This could assist efforts to establish citizen trust and consequently the mobilization of the real-world patient data for RWE. However, the provisions on data governance—which will largely influence data sharing—are in a more advanced stage of preparation, with the European Parliament aiming to adopt its position on the proposals during the summer of 2021. In parallel, several Member States are registering complaints about what they regard as excessive privacy safeguards, such as the requirement for repeat users to continually assess the risk of re-identification. For access to health data to inspire trust and be efficient, it is vital that the data governance provisions do not restrict options for the EHDS and provide adapted options depending on the type of RWD needing to be shared.

The importance of artificial intelligence (AI) for the processing of the big data underlying RWD—and of the trustworthy use of RWD to train AI—confers particular significance on discussions regarding RWE, which are still at an early stage in Europe. However, until RWD sets contain sufficiently large amounts of data, including biomarker and genomic data (which at present are quite rare) it is unclear what value AI would have and how it would be used. Promoting AI quality and safety standards while creating an ethical framework is another European Commission priority and health is one of the areas that has been highlighted since building a legislative framework began. The AI Act is now being carried through the EU’s legislative process and has triggered strong feelings and heated discussion regarding how best to protect privacy. Notably, a major concern with AI has been in relation to facial recognition applications and this has dominated the political narrative and has skewed discussions around the sharing of public health data. The success of AI relies on the availability of high-quality data for analysis; therefore, equal attention should be given to providing guidance on the issues that affect the quality of RWD that are outlined above.

The Pharmaceutical Strategy for Europe (adopted as a concept on 25 November 2020) aims to ensure access to affordable medicines, to support the development of competitive, innovative, and sustainable medicines, to ensure preparedness and response to health crises, and to promote quality, efficacy, and safety [21,23]. It is a patient-centered strategy that will be a key pillar of the European Commission’s aim to build a stronger EHU and will contribute to the implementation of other key action plans, e.g., the European strategy for data and the creation of the EHDS [21]. The specific rule changes envisaged under this strategy will not appear until later in 2022, and will cover issues as diverse as research incentives and obligations for the marketing of medicines. The wide scope of this measure—aiming to satisfy a number of overlapping and occasionally competing objectives, such as promoting innovation while ensuring access to medicines—carries the risk that without close attention from stakeholders it could result in lack of coherence in policy making. Here, also, the objectives would benefit from cross-EU and national–regional RWE.

The EU Cancer Mission is part of the Horizon Europe framework that aims to set common goals to tackle the burden of cancer across Europe [22]. With similar goals, the Europe’s Beating Cancer Plan aims to update the previous European action plan against cancer, taking into consideration the progress that has been made in cancer treatment over the last three decades and the EU Cancer Mission’s recommendations [20]. The plan outlines the actions that will support Member States’ responsibilities in healthcare and adds value for prevention, early detection, diagnosis and treatment, and improvement in quality of life for patients with cancer [20]. The need for data sharing and RWE has been stressed by all stakeholders. It is planned as another pillar of the EHU and could provide substantial financial support to Member States (potentially €4 billion) [20].

The Million European Genomes Alliance (MEGA) program—launched in 2018 and now the EU 1+ Million Genomes Initiative [19,20,24]—is a parallel initiative from health sector stakeholder groups across 22 EU countries, as well as the UK and Norway. It provides a potentially impactful model for building collaborations to share data, while at the same time inspiring trust among data owners as to how their data might be used. However, with the notable exception of this initiative—which should reach a goal of at least 1 million sequenced genomes accessible in the EU this year—the near future will tell how the genomes are shared and shown to provide RWE. As the European Commission stated in 2018: “It is paramount to agree on technical specifications for access and exchange of health data for research and public health purposes, addressing, for example, health data collection, storage, compression, processing, and access across the EU” [25].

These priorities underscore persisting disparities in the sharing of RWD across Europe. Some of these result from non-alignment between the multiple current EU initiatives outlined above. Others are more specifically linked to the inherent data infrastructure aggravated when RWD is considered. Differences in the way RWD are collected within countries may pose problems, e.g., the Italian healthcare system is divided into 20 regions and it can be difficult to implement consistent practices nationwide due to differences in standards/technology, etc. While GDPR provides a solid foundation of trust in the use of RWD in Europe, individual Member States have diverging interpretations—and therefore legislations and governance models—that could impact cross-country data sharing. Smaller countries, e.g., Bulgaria and Malta, may not be able to collect data using the same approach as larger countries due to a lack of opportunity, infrastructure, or capacity.

There is often a lack of transparency and consistency regarding the level of data that different stakeholders and regulatory authorities require. Resistance to RWD by European regulatory bodies is common, even when the same data are recognized by the US Food and Drug Administration (FDA). In Europe, data from traditional clinical trials are often expected to be available after the end of the clinical trial, including RWD. However, valuable data from EU-funded projects are at risk of not being collected because support often lacks vision of how to continue after the expiration of grant funding. They also suffer from duplication of effort, with different institutions or even different countries performing studies to answer the same scientific or medical questions.

As a result of the COVID-19 pandemic, the public is beginning to observe more tangible outputs from the RWD that they have contributed. This should be leveraged to improve public engagement in RWD collection. Public engagement around the use of their RWD could also become a more deliberate process through mission groups. Lessons from the COVID-19 pandemic could further be used to develop similar data collection systems for other diseases—such as cancer—to promote collection of real-time data outside of crisis situations like infectious diseases, and to provide examples of how national laws can be overcome to allow cross-border collaboration.

### 4.2. Recent and Upcoming Changes and Valuable Initiatives to Support RWE across Europe

A model to facilitate implementation of RWE at both country-specific and EU-wide levels is shown in Table 1. This model outlines frameworks for addressing four key challenges related to RWE uptake. Several initiatives—which already exist or are in development across Europe (Table 2)—aim to support implementation of RWE and can offer valuable insights into best practice that could be extrapolated to other settings.

#### 4.2.1. Public Trust and Access to Data

Recommendation: optimization of RWE implementation across Europe and transnational access to RWD in line with all applicable regulations and best practice.

National initiatives to enable access to data—such as Findata [26] in Finland—have been relatively successful. Findata is based on Finnish legislation regarding the secondary use of health data (Act on the Secondary Use of Health and Social Data [552/2019]) and is an authority that both provides permits for access to patient-level health data and delivers these data from different controllers to the permit holder [26].

At the European level—although there are numerous practical challenges associated with data sharing between countries—international data sharing is possible; as demonstrated by the Electronic Cross-Border Health Services [27]. This initiative from the European Commission already facilitates exchange of health data in the form of translated patient summaries and allows e-prescriptions to be filled between a select few European countries [27]. The aim is to implement this service across 25 EU countries by 2025 and to expand the patient data available to include medical images, laboratory results, and hospital discharge reports, and eventually full patient health records [27].

Transnational access to RWD is particularly important in the field of rare diseases where pan-European data access can help to solve challenges surrounding small population sizes and lack of power in clinical trials. Similarly, in oncology where the evolution of molecular tumor characterization has resulted in cancers originating from the same organ no longer being considered one single disease, similar principles to those used for studies of rare diseases can also be applied to molecularly defined subpopulations of patients with cancer.

Ensuring that data access is regulated and in line with best practice is essential for ascertaining citizen trust in cross-border data sharing. Despite the interest and growing understanding from patients on the use of their data to advance medical research, they may still have concerns over data security and ownership, and suspicion around sharing their data with governments or entities that appear to have their own vested interests.

**Table 2:** Initiatives in Europe to address the generation and use of RWE in healthcare.

Initiative	Country	Description
**Data Collection**		
European Cancer Patient Digital Centre	Europe	Facilitates the uptake of digital technologies to maximize their potential in cancer care [28]
RWE4Decisions	Europe	Brings together multiple stakeholders (policy makers, HTAs, payers, regulatory agencies, patient groups, academics, and industry) to decide which types of RWD could be collected for informing decisions by healthcare systems, clinicians, and patients [29]
Pan-Cancer Global Registry (industry led)	Global	Collection of health data from patients across the world with different cancers
**Enables Access to Data**		
Findata	Finland	Provides permits for access to patient-level data from different controllers and delivers these data to the user [26]
Health Data Hub	France	Covers the French National Health Data System (SNDS), which includes all the health data associated with a H-health insurance reimbursement from hospital treatments, doctors’ visits, participation in a research cohort, or an epidemiological/practice register [30]
Electronic Cross-Border Health Services	Select European countries but to be implemented across 25 EU countries by 2025 [27]	Allows sharing of patient summaries to doctors from other EU countries and permits pharmacists to dispense e-prescriptions to patients from other EU countries [27]
European Joint Programme on Rare Diseases (EJP RD)	26 EU Member States, UK, Canada, and seven associated countries (Armenia, Georgia, Israel, Norway, Serbia, Switzerland, and Turkey) [31]	Supports research into rare diseases by offering funding, pooling data resources and tools, educating researchers, and accelerating translation of results into effective treatments [31]
European Health Data Space (EHDS)	EU	One of the priorities of the European Commission 2019–2025 is to promote better access to data from different sources (electronic health records, genomics data, patient registry data, etc.) to support healthcare delivery and for use in research and health policy making. The system will address three main issues: strong data governance, data quality and interoperability, and strong infrastructure [32]
**Data Use/Evidence Generation**		
FinnGen	Finland	A study that combines genome information from Finnish biobanks with national healthcare registry data [33]
Drug Rediscovery Protocol (DRUP) study	The Netherlands	Assesses the efficacy and safety of commercially available, targeted anti-cancer drugs in patients with rare subgroups of cancers with actionable mutations [34]
Coverage with evidence development, e.g., the Cancer Drugs Fund	UK	Use of real-world data from interventions and relevant comparators from several sources to assess re-evaluation and funding of anti-cancer therapies [35]
GetReal Institute	Europe	Facilitates collaboration between RWE stakeholders to help to overcome challenges related to its generation and use, as well as providing a platform to assess and improve RWE quality and provide education on best practices [36]
European Initiative to Understand Cancer (UNCAN)	EU	Use of Europe-wide data to improve understanding of cancer risk, screening, diagnosis, treatment, quality of life, etc. [37]
**Data Collection/Data Use/Evidence Generation**		
National Health Service (NHS) Digital	UK	Organization responsible for managing and keeping national health data safe, as well as using it to improve understanding of health problems and to improve NHS services [38]
Data Analysis and Real World Interrogation Network (DARWIN) EU	EU	An EMA initiative, which aims to establish a catalog of high-quality, validated observational data that can be used in non-interventional studies to generate RWE to support regulatory decision-making [39]
**Policy Making**		
German Genomics Initiative (genomDE)	Germany	Promotes the introduction of genomic sequencing into routine healthcare for combination with other relevant health data to guide treatment decisions [40]
Genomic Medicine Sweden (GMS)	Sweden	Promotes collaboration between different stakeholders to allow effective utilization of advanced technologies for high-quality genomic testing in routine clinical practice [41]
**Engagement/Education**		
Innovative Partnership for Action Against Cancer (iPAAC) and Horizon 2020 Joint Action	24 European countries	Work Package 6—Genomics in Cancer Control and Care: Aims to engage and educate citizens/healthcare professionals/policy makers regarding issues on the use of genomic information in healthcare [42]

EMA, European Medicines Agency; EU, European Union; RWE, real-world evidence.

Engaging with and educating citizens around the use of health data can help to improve trust in data sharing for these purposes. For example, the Innovative Partnership for Action Against Cancer (iPAAC) Joint Action has an initiative to educate citizens, healthcare professionals, and policy makers about genomic testing in healthcare to increase its uptake in oncology [42]. Similar methods could be extrapolated for use in educating citizens around the necessity for and return on investment in data sharing.

Any multi-stakeholder healthcare system collaboration should support key actions to establish an agreed vocabulary specific to data to enable a shared understanding in the European Data Space. This could help support healthcare systems to effectively navigate complex data ecosystems beyond the traditional players to other sectors, such as information technology, environment, mobility, and finance sectors, and to establish data literacy for building trust in data overall. Educational programs for carers, highlighting the risks and values of sharing data, could improve the understanding for secure sharing of data and applying individual rights—which are key to a strong, trusted data ecosystem. Such education should extend beyond understanding of individual rights and responsibilities of the value that data sharing provides, to the potential insights and benefits generated through advanced diagnostics, AI, and machine learning. This could be supported by creating ethical frameworks, similar to the EU Guidelines on Ethics in artificial intelligence [43].

#### 4.2.2. Evidence Framework

Recommendation: alignment of RWE needs and sharing studies based on effective RWE methodologies across different countries to allow for incentivization.

Alignment of RWE needs would also ensure that investment for innovative healthcare is delivered on a global scale, with the potential to reduce costs for healthcare systems. Initiatives such as the German Genomics Initiative (genomDE) [40] and Genomic Medicine Sweden (GMS) [41] are involved in promoting the introduction of genomic testing into routine clinical practice in their respective countries as a method for improving prevention, diagnosis, and treatment of diseases. Implementation of similar initiatives in other countries would greatly increase the level of genomic testing and improve our understanding of the genetic associations of various diseases.

Germany also has an advanced framework for the assessment of Digital Health Applications (DiGA) designed to promote healthy lifestyles and for the detection or treatment of diseases [44]. The Federal Institute for Drugs and Medical Devices (BfArM) assesses these applications to verify qualities, such as data protection, interoperability, and user-friendliness, alongside reviewing evidence from the manufacturer relating to the effectiveness of the application [44]. Approved technologies are then added to the DiGA directory, which provides details to physicians on the features and effectiveness of the application, as well as information on prescription and reimbursement [44]. This method of assessing digital health applications may help to improve uptake of these technologies, thus increasing the potential of RWD collection in addition to improving the trust of regulatory agencies regarding these data.

#### 4.2.3. Cross-Border Governance Framework to Facilitate RWE Decision-Making

Recommendation: facilitation of early dialogue with regulators, HTAs, and payers, specifically to discuss the need for RWE in their decision-making frameworks.

Regulators have more experience of handling RWD, given their long history in the field of pharmacovigilance. While HTAs and payers have used RWD in economic modeling, both need to align to agree optimal approaches for the generation of RWE that can contribute to the determination of efficacy and effectiveness. This early dialogue with regulators, HTAs, and payers is essential to coordinate RWD collection requirements and optimize the use of patient data, and would help to clarify and align expectations for RWD between different stakeholders. Development of an RWE infrastructure with guidelines and standards based on common global standards would help to build regulators’ and payers’ trust in RWE, which is important considering the level of risk associated with the decisions that they are required to make.

Rare diseases are an appropriate example of how early dialogue can improve trust from regulatory bodies and facilitate changes in the types of evidence that are accepted outside of standard clinical trials. Studies into rare diseases have inherent methodological issues associated with the small patient populations involved; however, by establishing a dialogue and working closely with the EMA, the European Joint Programme on Rare Diseases (EJP RD) [31] has been able to develop common language and standards in rare disease clinical trials that allow use of the evidence generated in regulatory decision-making.

#### 4.2.4. Citizens—Improvements in Healthcare Decisions

Recommendation: enabling citizens to contribute to decision-making using their data.

The ultimate goal of optimizing implementation of RWE in healthcare is to improve quality of life for patients and citizens, by ensuring evidence-based and rational allocation of resources into disease prevention and early diagnosis, and refining treatment strategies in an effort to personalize treatment and improve patients’ quality of life and overall healthcare system efficiencies. Studies such as the Drug Rediscovery Protocol (DRUP) trial [34] can improve healthcare system efficiencies by assessing the efficacy and safety of commercially available, targeted anti-cancer therapies in rare subgroups of patients with actionable mutations. By using RWD to examine open-label use of drugs, treatment strategies for patients who may have limited therapeutic options may improve and information can be obtained for regulatory and reimbursement decisions for treatments offered outside of their approved use.

The UK’s Cancer Drugs Fund [35] uses outcomes data for interventions and any relevant comparators from several data sources, including the Systemic Anti-Cancer Therapy Dataset, Phase IV or pharmacovigilance studies, and tumor registries, to assess the effectiveness or cost-effectiveness of anti-cancer therapies. This system of ‘coverage with evidence development’ permits access to innovative drugs alongside the collection of RWD to inform a reassessment to determine whether a drug should be made routinely available. Similarly, in France, three administrative databases are now linked in the French Health Data Hub [30].

### 4.3. Key Recommendations to Support the Use of RWE-Based Studies across Europe

Several initiatives to improve the availability of RWD for evidence generation already exist in Europe and provide tangible examples of best practice or areas for development regarding the development and use of RWE. These examples have provided valuable lessons that can be extrapolated for use in other settings to allow future improvement of RWD collection, access, and use for healthcare decisions. In addition—and based on the discussions with our expert panel—we identified key recommendations aimed at addressing the barriers to implementation of RWE across Europe. These recommendations were:

- To develop consensus guidelines to standardize RWD generation and use. This should include best practice to shape methodology, analysis, and reporting of RWD and RWE, data sharing according to GDPR, and assessment of the clinical utility of RWD generation. Health data must be secure, provide a high level of quality, and ensure interoperability.
- The development of guidelines to allow consistent reporting of RWE-based studies in medical journals; few medical journals provide specific instructions for authors wishing to submit manuscripts on this type of study, leading to a lack of alignment in methods for conducting and reporting such data [45]. RWE-based studies that follow these guidelines should address payer and regulatory body concerns regarding the generation of RWE, thus aiding their incorporation into the value assessment pathway.

## 5. Conclusions

Our expert panel discussed a number of challenges facing the implementation of RWE across Europe, including methodological and data-quality issues, lack of harmonization between RWE data collection systems, data-access and data-sharing limitations, limitations of regulatory agencies or HTAs/payers, and lack of citizen trust in data sharing. Our recommendations to address these challenges should aid the routine European-wide implementation of RWE into health systems and health policy decision-making, and help physicians to realize the potential opportunities concerning the use of RWE in areas such as rare diseases and oncology. In addition, the EU 1+ Million Genomes Initiative could offer a useful model for advances toward healthcare system cooperation.

The necessary changes recommended in this article will not occur of their own volition; it will require strategic reflection and deliberate action to form the relevant connections. Focused efforts in EU policy making by those who recognize the need for change are a precondition to persuade those who have not identified the same requirements, particularly when they are gatekeepers within the health policy framework.

## Figures and Tables

**Table 1:** Maturity level model to address key issues affecting citizen trust and public engagement relating to the generation and use of RWE in healthcare.

#	Framework	Public Trust and Access to Data	Evidence Framework	Cross-Border Governance Framework to Facilitate RWE Decision-Making	Citizens—Improvements in Healthcare Decisions
5	Optimal	Transnational access to RWE according to applicable regulations and best practice guidance	Alignment of evidence needs to incentivize and ensure investment for innovative healthcare that can be delivered on a global scale and at a reduced cost for healthcare systems	Early dialogue with regulators, payers, and insurers, ad hoc infrastructure, guidelines, common global standards	Evidence exists to ensure rational allocation of resources for health and well-being; thus, improving healthcare system efficiencies
4	European	Governance framework for transnational protected access to quality-controlled data allows for evidence to be developed and accepted according to the applicable regulations and requirements across EU and Member States. Governance framework is aligned with regulator and payer requirements	A mechanism to communicate on the use of RWE to increase public trust with studies; showing how its use supports clinical and reimbursement decisions and promotes innovation across the EU. This would allow improved use of RWE in healthcare systems	A framework for RWE utilization agreed through an EU health governance framework that follows common standards and evidence requirements to facilitate decision-making and prioritization of evidence challenges by regulators and payers	Better prevention, diagnostic, and treatment decisions across the EU. Improvements in patient and citizen QoL. Healthcare actors, patients, and citizens call for framework utilization to support the best use of RWE
3	National	Alignment with EU regulations and prioritization of evidence challenges across Member States. Early dialogue with regulators/payers	Data from national clinical centers and research institutions are accessible for RWE decision makers	Governance framework that facilitates the federation of national infrastructure to enable collection, use, and interpretation of data for clinical and reimbursement decisions	Patients and citizens benefit from RWE being shared at the national level and there is a standard for evidence alignment
2	Regional /Local	Alignment with national regulations and inter-regional sharing of data for RWE	Data from federated regional clinical centers and research institutions are accessible for RWE decision makers	Regional infrastructure to reuse genomics and health data for RWE decision-making is lacking	Patients and citizens benefit from RWE being shared and utilized at the regional level
1	Elementary stage	Lack of alignment with regulation that prevents use of RWD or RWE. Lack of citizen trust leading to the absence of a system for RWE	Lack of methodology for an evidence framework to assess RWE. No agreed endpoints	No national infrastructure or governance available for adoption of RWE. No early dialogue between regulators and payers	No evidence is taken into consideration during patient diagnosis or treatment decision-making

EU, European Union; QoL, quality of life; RWD, real-world data; RWE, real-world evidence.

## Data Availability

Not applicable.

## References

[1] Hechenbleikner E.M., Samarov D.V., Lin E. (2020). Data explosion during COVID-19: A call for collaboration with the tech industry & data scrutiny. EClinicalMedicine.

[2] Cave A., Kurz X., Arlett P. (2019). Real-world data for regulatory decision making: Challenges and possible solutions for Europe. Clin. Pharmacol. Ther..

[3] Booth C.M., Karim S., Mackillop W.J. (2019). Real-world data: Towards achieving the achievable in cancer care. Nat. Rev. Clin. Oncol..

[4] Deverka P.A., Douglas M.P., Phillips K.A. (2020). Use of real-world evidence in us payer coverage decision-making for next-generation sequencing-based tests: Challenges, opportunities, and potential solutions. Value Health.

[5] Feinberg B.A., Gajra A., Zettler M.E., Phillips T.D., Phillips E.G., Kish J.K. (2020). Use of real-world evidence to support FDA approval of oncology drugs. Value Health.

[6] From Testing to Targeted Treatment Program (FT3). 3 Representative Working Groups to Co-Create and Deliver. https://www.fromtestingtotargetedtreatments.org/working-groups/.

[7] Kim H.-S., Lee S., Kim J.H. (2018). Real-world evidence versus randomized controlled trial: Clinical research based on electronic medical records. J. Korean Med. Sci..

[8] Naidoo P., Bouharati C., Rambiritch V., Jose N., Karamchand S., Chilton R., Leisegang R. (2021). Real-world evidence and product development: Opportunities, challenges and risk mitigation. Wien. Klin. Wochenschr..

[9] Wilkinson M.D., Dumontier M., Aalbersberg I.J., Appleton G., Axton M., Baak A., Blomberg N., Boiten J.W., da Silva Santos L.B., Bourne P.E. (2016). The FAIR Guiding Principles for scientific data management and stewardship. Sci. Data.

[10] Dickson D., Johnson J., Bergan R., Owens R., Subbiah V., Kurzrock R. (2020). The master observational trial: A new class of master protocol to advance precision medicine. Cell.

[11] Makady A., Ten Ham R., de Boer A., Hillege H., Klungel O., Goettsch W. (2017). GetReal Workpackage 1. Policies for use of real-world data in health technology assessment (HTA): A comparative study of six HTA agencies. Value Health.

[12] European Commission Regulation of the European Parliament and of the Council on Health Technology Assessment and Amending Directive 2011/24/EU. https://eur-lex.europa.eu/legal-content/EN/TXT/PDF/?uri=CELEX:52018PC0051&from=EN.

[13] European Commission European Health Union: Protecting the Health of Europeans and Collectively Responding to Cross-border Health Crises. https://ec.europa.eu/info/strategy/priorities-2019-2024/promoting-our-european-way-life/european-health-union_en.

[14] Franklin P.K. (2016). Public health within the EU policy space: A qualitative study of Organized Civil Society (OCS) and the Health in All Policies (HiAP) approach. Public Health.

[15] Negrouk A., Lacombe D., Meunier F. (2018). Diverging EU health regulations: The urgent need for co ordination and convergence. J. Cancer Policy.

[16] EUR-Lex Summaries of EU Legislation: Public Health. https://eur-lex.europa.eu/summary/chapter/29.html.

[17] European Union Supporting Public Health in Europe. https://europa.eu/european-union/topics/health_en.

[18] McKinsey & Company Personalized Medicines: The Path Forward. https://www.mckinsey.com/~/media/mckinsey/dotcom/client_service/pharma%20and%20medical%20products/pmp%20new/pdfs/mckinsey%20on%20personalized%20medicine%20march%202013.pdf.

[19] Horgan D., Kent A. (2017). EU health policy, coherence, stakeholder diversity and their impact on the EMA. Biomed. Hub..

[20] European Commission Communication from the Commission to the European Parliament and the Council: Europe’s Beating Cancer Plan. https://ec.europa.eu/health/sites/default/files/non_communicable_diseases/docs/eu_cancer-plan_en.pdf.

[21] European Commission Pharmaceutical Strategy for Europe. https://ec.europa.eu/health/medicinal-products/pharmaceutical-strategy-europe_en.

[22] European Commission Mission Area: Cancer. https://ec.europa.eu/info/research-and-innovation/funding/funding-opportunities/funding-programmes-and-open-calls/horizon-europe/missions-horizon-europe/cancer_en.

[23] European Commission EU Health Policy: Overview. https://ec.europa.eu/health/policies/overview_en.

[24] European Society for Medical Oncology (ESMO) Sixteen EU Member States Sign the Genomics Declaration [News Release]. https://www.esmo.org/oncology-news/archive/sixteen-eu-member-states-sign-the-genomics-declaration.

[25] European Commission Communication from the Commission to the European Parliament, the Council, the European Economic and Social Committee and the Committee of the Regions Empty on Enabling the Digital Transformation of Health and Care in the Digital Single Market; Empowering Citizens and Building a Healthier Society. https://eur-lex.europa.eu/legal-content/EN/TXT/HTML/?uri=CELEX:52018DC0233&rid=8.

[26] Findata—Health and Social Data Permit Authority Findata Website. https://findata.fi/en/.

[27] European Commission Electronic Cross-Border Health Services. https://ec.europa.eu/health/ehealth/electronic_crossborder_healthservices_en.

[28] European Cancer Organisation Digital Health Network. https://www.europeancancer.org/topic-networks/4:digital-health.html.

[29] RWE4Decisions RWE4Decisions Website. https://rwe4decisions.com/.

[30] Health Data Hub Health Data Hub FAQ. https://www.health-data-hub.fr/page/faq-english.

[31] European Joint Programme on Rare Diseases (EJP RD) What Is EJP RD?. Project Structure..

[32] European Commission European Health Data Space. https://ec.europa.eu/health/ehealth/dataspace_en.

[33] Institute for Molecular Medicine Finland (FIMM), University of Helsinki FinnGen Website. https://www.finngen.fi/en.

[34] Van der Velden D.L., Hoes L.R., van der Wijngaart H., van Berge Henegouwen J.M., van Werkhoven E., Roepman P., Schilsky R.L., de Leng W.W.J., Huitema A.D.R., Nuijen B. (2019). The Drug Rediscovery protocol facilitates the expanded use of existing anticancer drugs. Nature.

[35] NHS England Appraisal and Funding of Cancer Drugs from July 2016 (Including the New Cancer Drugs Fund): A New Deal for Patients, Taxpayers and Industry. https://www.england.nhs.uk/wp-content/uploads/2013/04/cdf-sop.pdf.

[36] GetReal Institute GetReal Institute Website. https://www.getreal-institute.org/.

[37] European Cancer Organisation Interim Report of the Mission Board for Cancer. https://www.europeancancer.org/policy/13-policy/11-interim-report-of-the-mission-board-for-cancer.

[38] NHS Digital Data. https://digital.nhs.uk/data.

[39] European Medicines Agency (EMA) Data Analysis and Real World Interrogation Network (DARWIN EU). https://www.ema.europa.eu/en/about-us/how-we-work/big-data/data-analysis-real-world-interrogation-network-darwin-eu.

[40] Federal Ministry of Health The German Genomics Initiative—GenomDE. https://www.bundesgesundheitsministerium.de/en/international/european-health-policy/genomde-en.html.

[41] Genomic Medicine Sweden (GMS) Sweden’s Ambitious National Collaboration for Genomic Medicine. https://genomicmedicine.se/en/.

[42] Innovative Partnership for Action Against Cancer (iPAAC) Work Package 6—Genomics in Cancer Control and Care. https://www.ipaac.eu/en/work-packages/wp6/.

[43] Madiega T., European Parliamentary Research Service (EPRS) EU Guidelines on Ethics in Artificial Intelligence: Context and Implementation. https://www.europarl.europa.eu/RegData/etudes/BRIE/2019/640163/EPRS_BRI(2019)640163_EN.pdf.

[44] Federal Institute for Drugs and Medical Devices (BfArM) DiGA Digital Health Applications. https://www.bfarm.de/EN/Medical-devices/Tasks/Digital-Health-Applications/_node.html.

[45] Oehrlein E.M., Graff J.S., Perfetto E.M., Mullins C.D., Dubois R.W., Anyanwu C., Onukwugha E. (2018). Peer-reviewed journal editors’ views on real-world evidence. Int. J. Technol. Assess. Health Care.

